# Research on the Effect of *Pediococcus pentosaceus* on *Salmonella enteritidis*-Infected Chicken

**DOI:** 10.1155/2020/6416451

**Published:** 2020-10-10

**Authors:** Dan Lan, XinYu Xun, YaoDong Hu, NianZhen Li, ChaoWu Yang, XiaoSong Jiang, YiPing Liu

**Affiliations:** ^1^Farm Animal Genetic Resources Exploration and Innovation Key Laboratory of Sichuan Province, Sichuan Agricultural University, Chengdu, Sichuan, China; ^2^Sichuan Animal Science Academy, Chengdu, Sichuan 610066, China

## Abstract

*Salmonella enteritidis* can cause significant morbidity and mortality in humans and economic loss in the animal industry. Improving the innate immunity is an effective method to prevent *S. enteritidis* infection. *Pediococcus pentosaceus* is a Gram-positive coccus which had probiotics properties. Numerous previously published studies reported that probiotics were beneficial to gut microbiota by changing the intestinal flora structure and inhibiting the harmful microbial growth to enhance the innate immunity. We investigated the immunological effects of *P. pentosaceus* on Salmonella-infected chickens by the following experiment. A total of 120 broilers from AA line were fed and divided into 2 groups (treated and control groups) for the experiment from day 1. The control group was fed with the basic diet, while the treated group was fed with the basic diet adding *P. pentosaceus* microcapsule with the bacterial concentration of 1 g/kg in the feed and bacterial counts 2.5 × 10^9^ CFU/g. All the birds were given with 0.5 ml of *S. enteritidis* bacterial suspension (10^9^ CFU/ml) through oral cavity at day 9. The number of dead birds was recorded and used in the analysis. The bacterial culture method and quantitative real-time PCR analysis were used to evaluate the effects of *P. pentosaceus* on chickens infected with *S. enteritidis* and to ascertain the mechanism of the effect. The results showed that the *P. pentosaceus* could restrain the pathogenicity of *S. enteritidis* and reduce the death rate from 44.4% to 23.3%. The flora in the caecum exhibited “rising-declining” trends, and the gene (*TLR4*, *MyD88*, *TRAF6 NF-κB*, *IFN-β*, *TNF-a*, *IL6*, and *IL8*) expression pattern was different between the experimental and control group. *P. pentosaceus* as a probiotic may competitively inhibit the growth of *S. enteritidis* and control the inflammatory response through regulating the gene expression which involved in the toll-like receptor pathway and inflammation pathway.

## 1. Introduction

*Salmonella enteritidis* is a common pathogenic bacterium for all species of mammals and fowls. *S. enteritidis* can cause serious economic loss in the animal industry, especially in the poultry production, and which also can influence human health [1]. Reports of worldwide human morbidity caused by infection with *S. enterica* started to appear as early as the mid-1970s [2], and the pathogenic factor most commonly associated with food like eggs [3]. Due to the pathogenicity and universality of *S. enteritidis*, the prevention is more important than treatment. Thus, improving the immunity of animals is an effective method to prevent infection.

*Pediococcus pentosaceus* (CGMCC No. 6566) is a Gram-positive coccus with probiotic properties [4]. An abundance of previous studies have reported that probiotics are beneficial to the gut microflora by changing the intestinal flora structure and inhibiting the harmful microbe growth in order to enhance the innate immunity [4–6]. Probiotics as an immunity activator can lead to production of antibodies and improve the phagocytic function of cells in order to stimulate the immune system, by inducing humoral immunity and cellular immunity, thus enhancing the resistance to diseases [7–11]. The *P. pentosaceus* bacterium used in this study was obtained from a Chinese indigenous Caoke chicken, as previous research had shown that *P. pentosaceus* can improve the meat quality of Caoke chicken. However, whether *P. pentosaceus* can improve the immunity of chicken needs to be further investigated.

Toll-like receptors (*TLRs*) are essential components of the innate immune system. To date, more than ten *TLR* genes have been found, including the toll-like receptor 4 (*TLR4)* gene which encodes an important factor for the innate immune system that senses bacterial lipopolysaccharide (*LPS*) and is a key player in the defense against pathogenic microorganisms [12]. Macrophages induce the innate immunity by recognizing pathogens through the *TLRs* that sense the pathogen-associated molecular patterns. The myeloid differentiation factor 88 (*MyD88*) encodes an essential adaptor protein molecule for most *TLRs* that mediate the induction of inflammatory cytokines through nuclear factor *κ*B (*NF-κB*) [13]. LPS can act through two different methods to activate the *TLR4/NF-κB* pathway; one is dependent on *MyD88*, whereas the other is not. Lomaga et al. [14] showed that *TNF* receptor-associated factor 6 (*TRAF6*) was crucial not only in interleukin 1 (IL-1) and *CD40* signaling, but also, surprisingly, in *LPS* signaling and was also essential for prenatal and postnatal survival. The immune response is a complex process involving the innate immune system, whose activation is indicated by the release of,inflammatory factors, such as *IFN-β*, *TNF-α*, *IL6*, and *IL8*, which play an important role in this process.

In this study, we aimed to investigate the effect of the *P. pentosaceus* on *S. enteritidis* infection. To this end, we evaluated the body condition change, caecum flora count, and the expression of *TLR* pathway genes and inflammatory factor genes in the spleen and caecum of *S. enteritidis-*infected chickens, which had been fed a diet with or without *P. pentosaceus*.

## 2. Materials and Methods

### 2.1. Birds and Management

A total of 120 arbor acre broilers (AA) were divided into 2 groups (treated and control groups) and fed for the experiment from day 1. The control group was fed the basic diet (composition and nutrient levels are shown in Table 1), while the treated group was fed the basic diet plus *P. pentosaceus* microcapsule (1 g/kg, bacterial counts 2.5 × 10^9^ CFU/g), which was formed according to the Ning's method [15]. All birds were reared cage free under standard conditions of temperature, humidity, and ventilation. The chickens had free access to feed and water during the entire rearing period. Birds were managed with due consideration to bird welfare. All procedures involving animals were approved by the Institutional Animal Care and Use Committee of the Sichuan Agriculture University (DK20134457). The experimental animals were anesthetized by intravenous injection of pentobarbital sodium at a dose of 40 mg/kg and euthanized by high cervical dislocation. Alleviated the suffering of experimental animals as much as possible during the experiment and cremated them centrally after the experiment.

### 2.2. Birds' Treatment and Sample Collection

All the birds were given with 0.5 ml of *S. enteritidis* bacterial suspension (10^9^ CFU/ml) through oral cavity at day 9. For each group, 4 birds per time (1-, 3-, 7-, and 14-day postinjection) were collected for evaluating and sample collection. Each birds' spleen and one of caecum were collected immediately after slaughtered and transferred to liquid nitrogen for RNA extraction. Another caecum was collected, and the contents were extracted for bacterial culturing. Body temperature was measured through the cloaca at 5 time points, 0- and 12-hour postinjection (hpi), and 1-, 3-, and 7-day postinjection (dpi).

### 2.3. Caecum Microbiota Culture and Count

The contents of the caecum (0.2 g) were added into 1.8 ml peptone and mixed. Then, the mixture was diluted to 10, 10^2^, 10^3^, 10^4^, and 10^5^ and cultured in media (Brilliant Green Agar for Salmonella and MacConkey medium for Escherichia coli). Escherichia coli was cultivated in a 37°C incubator for 24 h and identified as Gram-negative, while the Salmonella was cultivated at 37°C in an incubator for 18-24 h and identified by triple sugar iron agar.

### 2.4. Total RNA Isolation and cDNA Synthesis

Total RNA was isolated from the spleens and caecum (about 100 mg from each tissue sample) using Trizol reagent (Invitrogen Corp., Carlsbad, CA, USA) following the manufacturer's instructions. Total RNA concentration and purity were determined at A260, 280, and 230 nM using the NanoVue Plus spectrophotometer (GE Healthcare, Chicago, IL. USA), and RNA integrity was evaluated by agarose-formaldehyde electrophoresis.

The first strand cDNA was obtained using the ImProm-II Reverse Transcription System (TaKaRa Biotechnology Co. Ltd., Dalian, China). The reaction was performed in a volume of 40 *μ*L containing 8 *μ*L of 5× PrimerScript Buffer, 2 *μ*L of PrimerScript RT Enzyme Mix I, 1 *μ*L of 50 *μ*M Oligo dT forward and reverse primer, 2 *μ*L of 100 *μ*M Random hexamers, 4 *μ*L of total RNA (400 ng), and 22 *μ*L RNase-free dH_2_O. The reverse transcription (RT) reaction was performed at 37°C for 15 min with a final step of 85°C for 15 s and then stored at −20°C.

### 2.5. Real-Time Quantitative PCR Analysis of mRNA Expression

The expression levels of 8 chicken gene mRNAs at different stages of development in different tissues were measured by real-time quantitative PCR (qPCR). Expression of the chicken *β*-actin gene (GenBank accession number NM_205518) was used as an internal control. Primers were designed and synthesized by TaKaRa Biotechnology Inc. (Table 2). Specific amplification was confirmed by direct sequencing of the amplified fragments. The qPCR was carried out in a CFX96 qPCR system (Bio-Rad, Inc., Hercules, CA, USA) using the IQ SYBR Green SuperMix (Bio-Rad, Inc.) according to the manufacturer's instructions.

The cycling conditions consisted of an initial denaturation step of 2 min at 95°C, followed by 39 cycles of 5 s at 95°C, 30 s at 60°C, 30 s at 72°C, followed by a final extension period of 5 min at 72°C. A melting curve analysis was performed at a temperature of 65°C to 95°C, increasing at a rate of 0.5°C/s. The qPCR reaction was performed in a volume of 10 *μ*L, which included 5 *μ*L 2× SYBR Green SuperMix (Bio-Rad, Inc.), 1 *μ*L of 10× diluted cDNA, 0.4 *μ*L of forward and reverse primers (350 nM stocks), and 3.2 *μ*L nuclease-free H_2_O. Each assay was conducted in triplicate in 96-well plates (Bio-Rad, Inc.). A no template control (NTC) for each primer set was included in each run. The range of amplification efficiency of those genes and *β*-actin was from 95% to 105%.

### 2.6. Statistical Analyses

The 2^−∆∆Ct^ method of quantification [16] was used to calculate the gene expression values. The formula of ∆∆Ct was followed.

(1) $$\begin{array}{c} \Delta \Delta \text{Ct}=\left(\text{Ct},\text{target}-\text{Ct},\text{action}\right)\text{timex}-\left(\text{Ct},\text{target}-\text{Ct},\text{action}\right)\text{time}0. \end{array}$$

Using the GLM procedure of SAS 8.2 (SAS Institute Inc., Cary, NC, USA), we analyzed the differences in gene expression between groups and at different time points by ANOVA and define the significance level as *P* < 0.05.

## 3. Results

### 3.1. Body Temperature

**Figure 1:**
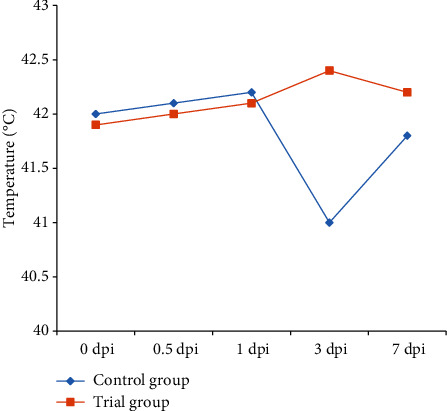
Comparison of body temperature postinfection between the two groups.

The results of the mean body temperature in the two groups were shown in Figure 1. Both groups had normal temperature at 0 hpi, whereas all animals had increased temperature after given with Salmonella. The control group had the highest temperature (42.2°C) at 1 dpi and then showed continuous decline, until it reached 41°C at 3 dpi. The experimental group experienced continuous rise in temperature during the first 3 dpi, when it reached up to 42.4°C. However, the temperature declined at 7 dpi, but it was still higher than it was before injection with Salmonella.

### 3.2. Health State and Death Rate

Most of the birds showed clinical symptoms at 12 hpi, which included depression, decreased ingestion, diarrheal disease, being afraid of the cold, and dyspnea. Those symptoms were more serious in the control group than in the experimental group. The infected chicken fed a diet without *P. pentosaceus* started to die at 1 dpi, and most of them died between 3 and 7 dpi, while no infected chickens fed a diet supplemented with *P. pentosaceus* had died at 7 dpi, and the birds became better. The death rates were 44.44% and 23.35%, in control group and experimental group, respectively. A dissection of a dead chicken that had shown symptoms like pericarditis, hepatomegaly, and hemorrhage is not shown.

### 3.3. Caecum Flora Count

The results of caecum bacterial count were shown in Table 3 and revealed that the flora in the caecum exhibited “rising-declining” trends. Specifically, after injection, the *Salmonella* number rose quickly and was highest at 3 dpi in the control group, whereas in the experimental group the increase in the number of *Salmonella* bacterium was gradual. Overall, the number of *Salmonella* in the control group was significantly higher than that in the experimental group.

The *Escherichia coli* number rose between 1 dpi and 3 dpi, and there was no significant difference between the two groups in the way in which the bacteria rose. Nevertheless, a comparison of the two groups revealed that the *Escherichia coli* in the control group was significantly (*P* < 0.05) higher than that in the experimental group at 1 and 7 dpi.

### 3.4. Gene Expression

**Figure 2:**
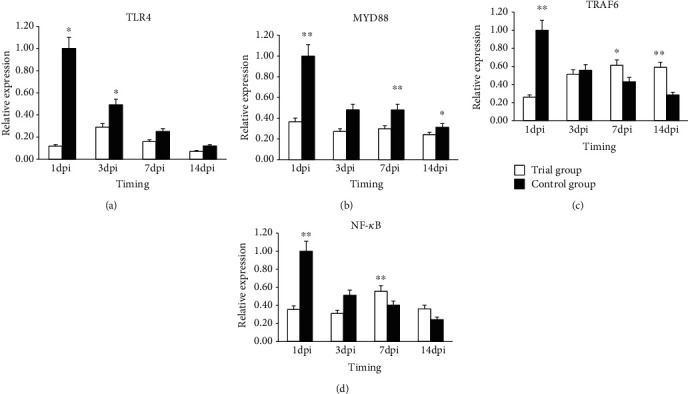
Expression of (a) *TLR4*, (b) *MyD88*, (c) *TRAF6*, and (d) *NF-κB* in the spleen samples of broiler chickens in the control and the treatment groups. Note: “^∗∗^” means the expression of the target gene between trial group and control group had highly significant (*P* < 0.01) difference; “^∗^” means the expression of the target gene between trial group and control group had significant (*P* < 0.05) differences. Error bars indicate the standard deviation.

The relative expression of 4 *TLR* pathway important genes (*TLR4*, *MyD88*, *TRAF6*, and *NF-κB*) and four inflammatory factor genes (*IFN-β*, *TNF-a*, *IL6*, and *IL8*) was analyzed in spleen and caecum at 4 different time points. Significant differences were detected between the control and experimental groups (Figure 2).

#### 3.4.1. Toll-Like Receptor Pathway Gene Expression in Spleen

In the control group, the relative expression of the *TLR4* gene was highest at 1dpi and subsequently declined at 3, 7, and 14 dpi, as shown in Figure 2(a). In the experimental group, the highest expression was at 3 dpi, and the gene expression exhibited a “rising-declining” trend after that. Overall, the expression of the *TLR4* gene was higher in the control group than that in the experimental group, and the difference was significant (*P* < 0.05) at 1 and 3 dpi.

The relative expression of the *MyD88* gene during the time it was evaluated exhibited a declining trend in both the control group and experimental group (Figure 2(b)). However, overall, the expression of this gene was higher in the control group than that in the experimental group, and there was a very significant difference (*P* < 0.01) at 1 and 7 dpi and a significant difference (*P* < 0.05) at 14 dpi.

The relative expression of the *TRAF6* gene showed a declining trend in the control group, which was highest at 1 dpi (Figure 2(c)). The experimental group was different from the control group, which was lowest at 1 dpi, but then continuously rose to the highest level at 7 dpi. The experimental group was lower than the control group at 1dpi, and the difference was highly significant (*P* < 0.01). The experimental group showed higher expression than the control group at 7 and 14 dpi, and the difference was significant (*P* < 0.05) and highly significant (*P* < 0.01), respectively.

The relative expression of the *NF-κB* gene exhibited a declining trend in the control group, which was highest at 1 dpi (Figure 2(d)). The experimental group showed a “declining-rising-declining” trend, which was highest at 7 dpi. Overall, the control group was higher than the experimental group at 1 dpi and 3 dpi, and the difference was very significant (*P* < 0.01).

#### 3.4.2. Toll Pathway Gene Expression in Caecum

**Figure 3:**
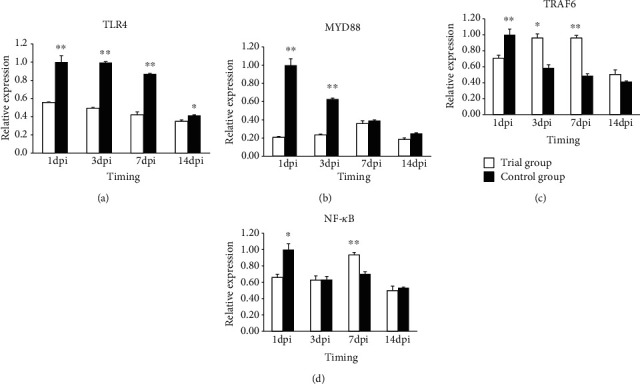
Expression of (a) *TLR4*, (b) *MyD88*, (c) *TRAF6*, and (d) *NF-κB* in the caecum samples of broiler chickens in the control group and the treatment group. Note: “^∗∗^” means the difference in the expression of the target gene between the experimental and control groups was highly significant (*P* < 0.01); “^∗^” means the difference in the expression of the target gene between the experimental and control groups was significant (*P* < 0.05). Error bars represent standard deviation.

The relative expression of the *TLR4* gene exhibited a declining trend both in the control and experimental group (Figure 3(a)), and the expression in the experimental group was highly significantly (*P* < 0.01) lower than that in the control group at the first three time points and significantly (*P* < 0.05) lower at 14 dpi.

The relative expression of the *MyD88* gene showed different trends between the control group and experimental group (Figure 3(b)). The control group exhibited a declining trend, which was highest at 1 dpi, and subsequently continued its decline. The trial group had a “rising-declining” trend and was highest at 7 dpi. A comparison of the two groups indicated that their difference was very significant (*P* < 0.01) at 1 dpi and 3 dpi.

The relative expression of the *TRAF6 g*ene showed different trends between the control and experimental groups (Figure 3(c)). The control group exhibited a declining trend, which was highest at 1 dpi, and continued to decline. The experimental group had a “rising-declining” trend, which was highest at 7 dpi, but the expression was not significantly different between 3 dpi and 7 dpi. A comparison of the two groups revealed that the control group had a lower expression than the experimental group, and the difference was significant (*P* < 0.05) and very significant (*P* < 0.01) at 3 dpi and 7 dpi, respectively.

The relative expression of the *NF-κB g*ene showed a different trend between the control and experimental groups (Figure 3(d)). The control group had a declining trend, and it was highest at 1 dpi and continued to decline. The experimental group exhibited a “rising-declining” trend and highest at 7 dpi. A comparison of the two groups revealed that the expression in the experimental group was significantly (*P* < 0.05) lower than that in the control group, which was very significantly (*P* < 0.01) higher than that in the control group at 7 dpi.

#### 3.4.3. Inflammatory Factor Gene Expression in Spleen

**Figure 4:**
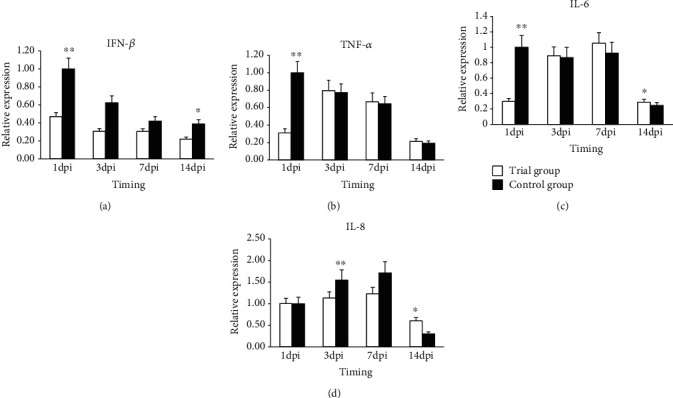
Expression of (a) *IFN-β*, (b) *TNF-a*, (c) *IL6*, and (d) *IL-8* genes in the spleen samples of broiler chickens in the control group and the treatment group. Note: “^∗∗^” means the difference in the expression of the target gene between the experimental and control groups was highly significant (*P* < 0.01); “^∗^” means the difference in the expression of the target gene between the experimental and control groups was significant (*P* < 0.05). Error bars represent standard deviation.

The relative expression of the *IFN-β g*ene showed a declining trend both in the control and experimental groups (Figure 4(a)). The control group always showed a higher expression than the experimental group, and the difference was highly significant (*P* < 0.01) and significant (*P* < 0.05) at 1 dpi and 14 dpi, respectively.

The relative expression of the *TNF-a g*ene exhibited different trends in the control and the experimental groups (Figure 4(b)). The control group had a declining trend, which was highest at 1 dpi, and continued to decline. The experimental group had a “rising-declining” trend, which was highest at 3 dpi. Expression in the experimental group was higher than in the control group, and the difference was not significant (*P* > 0.05) at 3, 7, and 14 dpi.

The relative expression of the *IL6* gene was highest at 1dpi in the control group (Figure 4(c)), and it was very significantly (*P* < 0.01) higher than that in the experimental group. The expression exhibited a rising trend in the experimental group, which was highest at 7 dpi. A comparison of the two groups revealed that the expression was always higher in the experimental group than that in the control group, and the difference reached significance (*P* < 0.05) at 14 dpi.

The relative expression of the *IL8* gene showed the same trend in the control and experimental groups (Figure 4(d)). The expression in both groups reached the highest levels at 7 and 14 dpi and subsequently declined to a level lower than that at 1 dpi. A comparison of the two groups revealed that there was no difference in expression at 1 dpi, while the expression was higher in the control group than that in the experimental group at 3 and 7 dpi, and the difference was significant at 7 dpi. Remarkably, the expression was higher in the experimental group than in the control group at 14 dpi, and the difference was highly significant (*P* < 0.01).

#### 3.4.4. Inflammatory Factor Gene Express in Caecum

**Figure 5:**
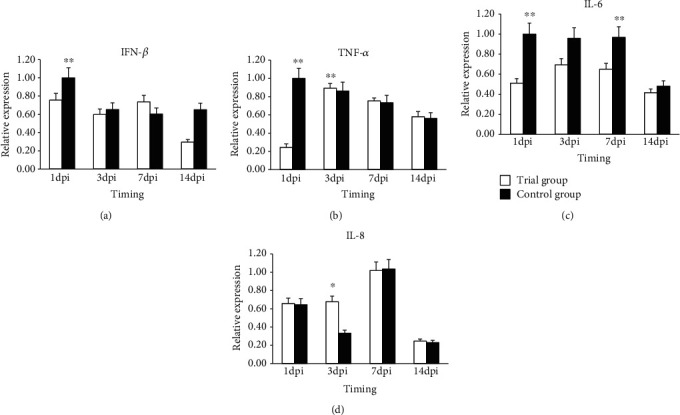
Expression of (a) *IFN-β*, (b) *TNF-a*, (c) *IL6*, and (d) *IL-8* genes in the caecum samples of broiler chickens in the control group and the treatment group. Note: “^∗∗^” means the difference in the expression of the target gene between the experimental and control groups was highly significant (*P* < 0.01); “^∗^” means the difference in expression of target gene between the experimental and control groups was significant (*P* < 0.05). Error bars represent standard deviation.

The relative expression of the *IFN-β* gene was highest at 1 dpi in both groups, but in the control group, it was very significantly (*P* < 0.01) higher than that in the experimental group (Figure 5(a)). The expression continuously declined in the control group, while it exhibited a declining-rising-declining trend at 3, 7, and 14 dpi, which was lowest at 14 dpi. A comparison of the two groups at all-time points, except at 7 dpi, revealed that the experimental group had higher expression than the control group.

The relative expression of the *TNF-a* gene showed a declining trend in the control group and was highest at 1 dpi, which was very significantly (*P* < 0.01) higher than that in the experimental group (Figure 5(b)). The experimental group had the highest expression at 3 dpi and then declined at 7 and 14 dpi. The expression in the experimental group was higher than that in the control group at 3 and 7 dpi, and the difference was very significant (*P* < 0.01) at 3 dpi.

The relative expression of the *IL6* gene was highest at 1 dpi in the control group, and it was significantly higher than that in the experimental group at 1 and 3 dpi. The expression was highest at 7 dpi in the experimental group and declined in 14 dpi, which was lower than that at 1 dpi (Figure 5(c)).

The relative expression of the *IL8* gene exhibited the same trend in both groups (Figure 5(d)). Both groups reached the highest expression at 7 dpi, and the expression in the experimental group was higher than that in the control group at 1, 3, and 7 dpi, and the difference was significant (*P* < 0.05) at 3 dpi.

### 3.5. Gene Expression in Difference Tissues

To further determine whether the expression of the above genes was tissue specific, we analyzed the expression levels of those genes in two different tissues. The results showed that the expression of four *TLR* pathway genes was “decreased” in both the spleen and caecum tissues in the control group, and the highest expression was at 1 dpi. The expression was different in the experimental group. Among the four inflammatory factor genes, expression of *IFN-β*, *TNF-a*, and *IL6* genes exhibited the same trends in those two tissues as over the time period at which they were evaluated, while the *IL8* gene showed different trends in the tissues examined.

## 4. Discussions

### 4.1. Body Temperature and Flora Number

After injection with the Salmonella bacterium, the body temperature rose in both groups. As the time progressed, the health of the birds in the control group deteriorated, the body temperature declined, and the number of dead birds rose. The rise in the body temperature was beneficial for developing resistance to the disease, whereas the low temperature was not beneficial to the enhancement of the inflammatory reaction whereby germs are killed. As the chicken was infected by Salmonella, the organism mounted an inflammatory response to kill germs, which led to a rise in the body temperature.

The death of chickens in the control group mainly occurred between 3 and 7 dpi, and the body temperature was lower than that of animals in the experimental group. This result was consistent with those of previous research. For instance, Liping and Yujie [17] reported that the broiler chicken fed a diet supplemented with complex microorganisms can improve the resistance to *S. pullorum* and reduce the chicken death rate to 20%. Additional studies reported that lactobacillus can improve the immune function and enhance the natural immunity and intestinal mucosa resistance [18–20]. We can speculate that continuous supplementation of the diet with *P. pentosaceus* can reduce the harmful effects of pathogenic bacteria in chicken.

Several studies have reported that probiotics can compete with *Salmonella* and *Escherichia Coli* to bind with Caco-2 to inhibit the pathogenic bacterium growth [21–23], and the microbial community structure was altered as the *E. coli* number increased when infected by *S. pullorum* [18]. Our study results on flora number revealed that the flora number in caecum had increased but not significantly and quickly returned to normal levels in the experimental group. Meanwhile, in the control group, the flora number quickly increased and the difference was significant. This result is consistent with those of the other studies and indicates that the infection with the pathogenic bacterium can change the microbial community structure in caecum, and the effect was less intense in the experimental group than that in the control group. It is possible that this may be caused by physical and mechanical competitive inhibition of the probiotics to reduce the Salmonella seeding. Additional studies indicated that *Lactobacillus*, as one of the probiotics, can secrete organic acid to reduce the pH value, produce secondary metabolites like bacteriostatic toxin, and inhibit the pathogenic bacterium growth to protect the intestinal biological barrier [24, 25]. *P. pentosaceus* which belongs to the *Lactobacillaceae* family may be share the same resistance mechanism.

### 4.2. Gene Expression

The *TLR* pathway plays an important role in mediating *Salmonella* transmembrane signal transduction and stimulates the body immune system. *TLR4*, *MyD88*, *TRAF6*, and *NF-κB* are important genes in the *TLR* pathway, and their expression can indicate the activation intensity of the *TLR* pathway. After injection with Salmonella, the gene expression changed along with time, which was consistent with previously reported results [26–29], indicating that the *TRL4* pathway was connected with the *S. enteritidis* infection. A comparison of the experimental and control groups revealed that the gene expression patterns of the two groups were not consistent, which indicated that the added *P. pentosaceus* in the diet can affect the gene expression. The *TLR4* and *MyD88* gene expression in spleen and caecum were higher in the control group than that in the experimental group. These results suggested that the pathogenic bacterium activated the *TLR* pathway, which was higher in the control group than that in the experimental group. This activation may occur as a result of the *P. pentosaceus*' competitive inhibition, physical, and mechanical that decreases the *Salmonella* seeding thus reducing the stimulation of intestinal cells by *LPS* to attenuate the immune response. Research has shown that *TLR4* expression in enterocyte was blocked, which contributes to maintaining the steady state [30]. Higher expression of *NF-κB* can increase the inflammatory reaction through activation of the downstream inflammatory factors in the *TLR4* pathway. *TNF-a* as an important inflammatory factor that can act as an anti-infection pyrogenic factor that causes fever. *IL6* can strengthen the effects of the other cytokines. The expression of inflammatory factor genes declined at 3 d, a result that was consistent with the body temperature change trend. According to our results, we can speculate that *P. pentosaceus* can restrain the activation of the TLR4 pathway by *S. enteritidis*, thus causing a reduction of *TLR4* signaling, a decline of the expression of the *MyD88* gene, and the decline of the expression of downstream inflammation factor genes. Whether the change in the expression of those genes caused the protein changes needs to be further investigated in the future. Why the expression of these genes declined between 3 dpi and 7 dpi in the control group, while they were increased in the experimental group? According to the results of the evaluation of the body temperature and flora number change and chicken death condition, we surmised that the decline in body temperature was not a contributing factor to the inflammatory response, which may have allowed the bacteria to grow and cause the rise in microbial load and the increased death rate.

In this study, we just comparatively analyzed the effect of added and not added *Pediococcus pentosaceus* on *Salmonella enteritidis*-infected chicken, so we do not design the healthy control group, and this is a bug. On this basis, it seems obvious that further study is needed to design the healthy control group.

## 5. Conclusions

Through this research, we can speculate that the *P .pentosaceus* can not completely eliminate the *S. enteritidis* infection and it just inhibited the bacterial growth. The mechanism of inhibition of *S. enteritidis* by *P. pentosaceus* may involve the inhibition of the expression of certain genes usually induced by *S. enteritidis* or alternatively may result from direct effects on the TLR4 gene and inhibition of its expression.

## Figures and Tables

**Table 1:** Composition and nutrient levels of basic diet.

Composition	Content (%)
Corn	55.18
Soybean meal	24.90
Fermentation protein	5
Compound oil	4
Wheat bran	4
Rapeseed meal	2.65
Calcium bicarbonate	1.79
Calcium carbonate	0.87
Mineral premix^1^	0.50
DL-methionine	0.16
Choline	0.10
Mildew preventive	0.10
Vitamin premix^2^	0.03
Salt	0.40
Bentonite	0.30
Nutrient levels	Content (%)
Crude protein	19.24
Crude cellulose	3.08
ME (MCal/kg of DM)	2.72
Lysine	1.05
Available phosphorous	0.21
Dicalcium phosphate	1.88
Calcium carbonate	0.91
Methionine	0.17

**Table 2:** Sequences of primers used for qPCR analysis.

Genes	Primer sequence (5′⟶3′)	Product size	GenBank ID
*β-Actin*	F:GCCAACAGAGAGAAGATGACAC R:GTAACACCATCACCAGAGTCCA	140	NM205518.1
*TLR4*	F:AGTCTGAAATTGCTGAGCTCAAAT R:GCGACGTTAAGCCATGGAAG	190	AY064697
*MyD88*	F:ACCTGGAAAGTGATGAATGT R:TTGTAATGAACCGCAAGATA	138	NC006089
*TRAF6*	F:ATGGAAGCCAAGCCAGAGTT R:ACAGCGCACCAGAAGGGTAT	144	NC006092
*NF-κB*	F:TCAACGCAGGACCTAAAGACAT R:GCAGATAGCCAAGTTCAGGATG	162	NM205134
*TNF-a*	F:CCGTAGTGCTGTTCTATGACCG R:GTTCCACATCTTTCAGAGCATCAA	189	NM204267.1
*IFN-β*	F:CCTCAACCAGATCCAGCATTAC R:CCCAGGTACAAGCACTGTAGTT	157	NM001024836
*IL6*	F:CGTTTATGGAGAAGACCGTGAG R:AGAGGATTGTGCCCGAACTAA	134	NM204628
*IL-8*	F:CTCAATTCTGATGCACCAC R:AATTAACATGAGGCACCGAT	105	NM205498

**Table 3:** Comparison of *Salmonella and Escherichia coli* load in the caecum of postinfection between the two groups of chicken (mean ± SD log10).

Strain	Group	Sample size	1 d	3 d	7 d	14 d
*Salmonella*	Control group	4	7.44 ± 0.42^b,x^	8.00 ± 0.33^a,y^	7.86 ± 0.16^a,y^	7.77 ± 0.31^a,y^
	Trial group	4	7.20 ± 0.18^a,x^	7.27 ± 0.69^a,x^	7.39 ± 0.20^a,x^	6.65 ± 0.42^b,x^
*Escherichia coli*	Control group	4	8.89 ± 0.09^a,y^	9.76 ± 0.40^a,y^	9.77 ± 0.45^a,y^	8.96 ± 0.34^a,x^
	Trial group	4	7.63 ± 0.35^a,x^	8.42 ± 0.42^a,x^	8.83 ± 0.39^a,x^	8.37 ± 0.41^a,x^

Note: ^a-b^ represent the comparison of the data in the group, and different superscripts mean significant difference (*P* < 0.05); ^x-y^ represent the comparison of the data between groups, and different superscripts mean significant difference (*P* < 0.05).

## Data Availability

Data supporting the findings is contained within the manuscript.

## Abbreviations

*S. enteritidis*: Salmonella enteritidis
*P. pentosaceus*: Pediococcus pentosaceus
*TLRs*: Toll-like receptors
*TLR4*: Toll-like receptor 4
*LPS*: Lippolysaccharide
*MyD88*: Myeloid differentiation factor 88
*NF-κB*: Nuclear factor *κ*B
*TRAF6*: TNF receptor-associated factor 6.
